# Efficacy and Safety of Human Placental Extract Solution on Fatigue: A Double-Blind, Randomized, Placebo-Controlled Study

**DOI:** 10.1155/2012/130875

**Published:** 2011-09-28

**Authors:** Kang-Kon Lee, Whan-Seok Choi, Keun-Sang Yum, Sang-Wook Song, Sun-Myeong Ock, Sat-Byul Park, Moon-Jong Kim

**Affiliations:** ^1^Department of Family Medicine, The Catholic University of Korea School of Medicine, Seocho-gu 137-701, Republic of Korea; ^2^Department of Family Medicine, Seoul St. Mary's Hospital, The Catholic University of Korea College of Medicine, 505 Banpo-dong, Seocho-gu 137-701, Republic of Korea; ^3^Department of Family Practice and Community Health, Ajou University School of Medicine, Suwon 443-721, Republic of Korea; ^4^Department of Family Medicine, Pundang CHA General Hospital, College of Medicine, Pochun Chung-mun University, Seongnam, Republic of Korea

## Abstract

*Introduction*. Fatigue is a common symptom, but only a few effective treatments are available. This study was conducted to assess the efficacy and safety of the human placental extract solution, which has been known to have a fatigue recovery effect. *Methods*. A total of 315 subjects were randomly assigned to three groups: group 1 (with Unicenta solution administration), group 2 (with exclusively human placental extract administration, excluding other ingredients from the Unicenta solution), and the placebo group. Subsequently, solutions were administered for four weeks. *Results*. The fatigue recovery rate was 71.00% in group 1, 71.72% in group 2, and 44.21% in the placebo group, which show statistically significant differences between the group 1 and the placebo group (*P* value = 0.0002), and between group 2 and the placebo group (*P* value = 0.0001). *Conclusion*. The human placental extract solution was effective in the improvement of fatigue.

## 1. Introduction

The placenta is an organ that stores many active molecules such as various nutrients and immune chemicals that are required for life sustenance and proliferation of fetus. Human placental extract (HPE) has been known to have many ingredients with biological activity and various curing effects. So far, molecules, such as hormones, proteins, lipids, nucleic acids, glycosaminoglycan, amino acids, vitamins, and minerals, have been extracted and identified from HPE [1–3]. In addition to a Japanese study that reported that the placenta extract was effective in recovery from fatigue and in ergogenic aid by increasing the blood flow to facilitate nutrition supply and promote excretion of accumulated body waste [4], several studies on antioxidation [2], anti-inflammation [4], and the whitening function [5, 6] have been conducted. Most of these studies, however, had a restriction in that they were conducted on animals or were not comprehensive. 

The history of use of the placenta as a curing agent is very old. Hippocrates in Greece used the placenta for treatments, and medical information on the placenta was included in many Chinese medical books. 

In modern medicine, Dr. Filatov of the Soviet Union first used the placenta to treat diseases in the 1930s. In Japan, treatments using the human placenta began in the 1950s, with indications of improvement of the hepatic function and menopausal disorder.

In South Korea, the placenta has been used in skin care and for recovery from fatigue or allergy in many clinics. The efficacy of the placenta has not yet been scientifically verified, though. Furthermore, its pharmaceutical mechanism and adverse events have not been fully studied yet. 

Although chronic fatigue is a common symptom in the primary medical care system, its causes are not clear in many cases, and effective treatment methods have not been well established. 

In this study, a multicenter, double-blind, randomized experiment was conducted using human placental extracts solution(Unicenta), which has been approved as having the effects of fatigue recovery and ergogenic aid, to evaluate the efficacy of the placenta on fatigue via score changes in the checklist of individual strength (CIS). In addition, the safety of the placenta was investigated by analyzing adverse events, vital signs, and laboratory test results. 

## 2. Materials and Methods

### 2.1. Study Design

Of the patients who visited one of six medical centers in Korea from September 10, 2008 to March 12, 2009, those who had persistent fatigue for one month or more were selected as the subjects of this study. 

The subject inclusion and exclusion criteria were as follows. 

#### 2.1.1. Inclusion Criteria

1. Age: 18–75 years,
2. persistent fatigue for one month or higher upon screening,
3. fatigue neither from organopathy nor continued exercise,
4. total CIS score ≥ 76 points upon screening,
5. Hospital Anxiety and Depression Scale (HADS) score ≤ 10 points upon screening.

#### 2.1.2. Exclusion Criteria

1. Previous record of alcohol or drug abuse,
2. hepatic and renal dysfunction (two times more than the normal upper limit in any of the followings: AST, ALT, total bilirubin, and blood creatinine),
3. chronic viral hepatitis B or C,
4. if the following underlying diseases were identified: uncontrolled hypertension (≥170/110 mmHg), diabetes, malignant tumor, tuberculosis, asthma, glaucoma, multiple sclerosis, hypothyroidism, major depressive disorder, bipolar disorder, schizophrenia, delusional disorder, dementia, bulimia nervosa, severe obesity (≥45 BMI), fatty liver, hepatitis due to medication/alcohol, liver cirrhosis, chronic renal failure, patient on dialysis, phenylketonuria,
5. investigators adjudged the difficulty of the patient's participation due to severe metabolic diseases, cardiovascular/cerebrovascular diseases, systemic infectious diseases, and gastrointestinal diseases that may cause physical and mental fatigue,
6. pregnancy, breast feeding, or women of childbearing age without a suitable contraceptive,
7. previous record of hypersensitivity to HPE or other animal derivatives,
8. exercise therapy or chemotherapy that should not be used in combination with study medication is expected to be conducted during the study period,
9. anti-HIV positive,
10. use of testosterone.

Of the 347 applicants who underwent the screening process, 32 subjects were excluded (not eligible for inclusion/exclusion criteria: 23, withdrawal of informed consent: 8, follow-up lost: 1). A total of 315 subjects were randomly assigned to three groups: Group 1 (with Unicenta solution administration), group 2 (with exclusively human placental extracts and excluding other ingredients from the Unicenta solution), and the placebo group. As the Unicenta solution includes other components (thiamin nitric acid, riboflavin sodium phosphate, pyridoxine hydrochloric acid, nicotinamide, and caffeine anhydrous) that have a fatigue recovery effect besides the human placental extract, the experimental groups were divided into two groups to assess the efficacy of the human placental extract by excluding other components that may affect fatigue recovery. 

Of the 315 subjects, 294 subjects completed this study, as 16 subjects were withdrawn due to their violation of the inclusion/exclusion criteria and five subjects did not undergo efficacy assessment. The subjects were instructed not to take other medications that may cause fatigue during the study period. 

All the subjects completed an informed consent form before participating in the study, and the study was conducted after the approval of the Institutional Review Board (IRB) of every participated medical centers.

### 2.2. Methods

After the subjects' completion of an informed consent form, their medical records, laboratory test results, and CIS [7] and HADS score [8] were reviewed, and their serious life events were assessed. The subjects took the assigned solutions once everyday for four weeks. On the second week of the study period their physical condition, vital signs, fatigue, serious life events, and adverse events were checked. Their laboratory test results, pregnancy test results, fatigue, serious life events, and adverse events were checked after the completion of the four-week study period. The subjects who had adverse events were checked again on the sixth week of the study period (Table 1). 

The subjects' improvement of fatigue was checked via CIS [7]. The questionnaire had 20 items, and a maximum of 7 points was given to each item. Higher total scores reflected a higher degree of fatigue. In this study, the criterion of the CIS score for the subject selection was 76 points. 

### 2.3. Statistical Analysis

SPSS for Windows Version 15.0 (SPSS Inc., USA) was used for the statistical analysis. The chi-square test or Fisher's exact test was conducted to investigate the difference in the frequency of the subjects who had improved CIS scores of less than 76 points which was found among the subject groups. In addition, the unpaired *t*-test was conducted to investigate the difference between the CIS score change at the baseline and on the fourth week was found among the subject groups. The chi-square test or Fisher's exact test was conducted to investigate the difference in the frequency of the subjects who had CIS score changes of 12 points or higher at the baseline and on the fourth week was found among the subject groups. As for the safety assessment of the human placental extract, the chi-square test or Fisher's exact test was conducted to investigate the difference in the adverse event rate which was found among the subject groups. All the data were expressed in the form of the mean ± standard deviation. The analysis results were considered statistically significant if the *P* value was < 0.05. 

## 3. Results

### 3.1. Demographic Information and Characteristics of the Subjects

As for the demographic characteristics of the subjects prior to the study, no statistically significant differences in all the HADS scores, except for the anxiety score, were found among the three groups. As a result of the multiple comparison, using Tukey's procedure, of the HADS anxiety scores that significantly differed among the subject groups, a statistically significant difference in the anxiety scores was found between the experimental groups 1 and 2. Therefore, it was presumed that the anxiety score difference would not affect the comparison of group 1 with the placebo group, and the comparison of group 2 with the placebo group, when the primary efficacy assessment was analyzed with the CIS recovery rate (Table 2). 

2.97% (3/101 subjects) of Group 1, 6.86% (7/102 subjects) of Group 2, and 2.08% (2/96 subjects) of the Placebo Group experienced serious life events such as death of a spouse, divorce or separation, death of close relatives, injury or disease of less than two weeks, marriage or engagement, school entrance, graduation, transfer, or movement. No significant difference was found, however, among the three groups (*P* value = 0.2544). 

### 3.2. Efficacy Assessment

#### 3.2.1. Fatigue Recovery Rate of the CIS Score

CIS, which is a tool for fatigue assessment, is used to measure the degree of fatigue of an individual. A lower CIS score means greater recovery from fatigue.

At visit 3, the fatigue recovery rate was 51.00% (51/100 subjects) in group 1 and 46.32% (44/95 subjects) in the placebo group. The fatigue recovery rate was higher in group 1 than in the placebo group, but no statistical difference was found between the two groups (*P *value = 0.5130). The fatigue recovery rate was higher in group 2 [58.59% (58/99 subjects)] than in the placebo group, but no statistical difference was found between the two groups (*P *value = 0.0871). At visit 4, the fatigue recovery rate was 71.00% (71/100 subjects) in group 1 and 44.21% (42/95 subjects) in the placebo group, which show a statistically significant difference (*P *value = 0.0002). The fatigue recovery rate was higher in group 2 [71.72% (71/99 subjects)] than in the placebo group, and the rates had a statistically significant difference (*P *value = 0.0001) (Table 3). 

#### 3.2.2. CIS Score Change

The CIS score measured at visit 4 decreased by 24.79 points (±15.27) in group 1, by 24.79 points (±15.27) in group 2, and by 17.12 points (±17.23) in the placebo group compared to the CIS scores at the baseline. The CIS score decreased with statistical significance in all the groups four weeks after the administration of study solution (*P *value < 0.0001). The difference in the CIS score changes between visit 4 and the baseline was statistically significant between group 1 and the placebo group (*P *value = 0.0012). The difference in the CIS score changes between visit 4 and the baseline was also statistically significant between group 2 and the placebo group (*P *value = 0.0039). The CIS score decreased more in the experimental groups 1 and 2 than in the placebo group (Table 4). 

#### 3.2.3. Rate of the CIS Score Reduction

The percentages of the subjects who had a reduced CIS score of more than 12 points at visit 4 over the baseline (visit 1) were 80.00% (80/100 subjects) in group 1 and 62.11% (59/9 subjects) in the placebo group, which show a statistically significant difference (*P *value = 0.0058). The percentage of the subjects who had a reduced CIS score of more than 12 points at visit 4 over the baseline (visit 1) was 76.77% (76/99 subjects) in group 2, which shows a statistically significant difference from that of the placebo group (*P *value = 0.0265) (Table 5). 

### 3.3. Safety Assessment

No statistically significant difference in the percentages of the subjects who experienced adverse events was found among the groups (*P *value = 0.9201). In this clinical trial, the percentage of the subjects who experienced serious adverse events was 0.99% (1/101 subjects, 1 case) in group 1. Such percentage turned out to be irrelevant with the study medication, though. No statistically significant difference in the ADR expressions was found among the groups (*P *value = 0.3477) (Table 6). 

As for the adverse events that occurred most frequently, acute pharyngitis and dyspepsia accounted for 2.97% (3 cases) and 2.97% (3 cases), respectively, in group 1. In addition, nausea accounted for 1.98% (2 cases). On the other hand, acute pharyngitis accounted for 3.92% (4 cases), which was the highest frequency in group 2. 

In addition, abdominal pain, headache, and urticaria accounted for 2.94% (3 cases), 1.96% (2 cases), and 1.96% (2 cases), respectively. Liver enzyme increase accounted for 3.13% (3 cases), which was the highest frequency in the placebo group. In addition, dyspepsia, acute pharyngitis, upper respiratory infection, and myalgia each accounted for 2.08% (2 cases). 

## 4. Discussion

Fatigue, which is an expression of a individual health condition, is a frequently occurring symptom that is associated with health problems. In most cases, it is temporary and improved by appropriate rest. If fatigue is severe or has compounded, however, it may weaken a person's capability to lead an ordinary life or cause various diseases, which will lead to reduced quality of life [9]. 

It is difficult to conduct a study on fatigue because fatigue is hard to assess objectively. Although the CIS score is commonly used in studies on fatigue, it does not resolve the aforementioned problem. Despite the statistical significance of the results of this study, difficulties in clinical studies on fatigue were seen because the placebo group had a fatigue recovery rate of more than 40%. The results of this study showed a relatively higher placebo effect than the results of other studies. This is likely to be due to the following reasons. First, the subjects could have participated in this study with high expectations from the “placental nutrients” that are sold at high prices. Second, the placebo used in this study had a sweet taste that was similar to that of the ergogenic drinks that are sold in the market, which could have made the subjects feel temporary recovery from psychological fatigue. 

This study has another disadvantage: the short duration of the administration period. Considering that the effect of the human placental extract does not emanate from its direct administration but from its interaction with cytokine, hormones, or other unknown ingredients in the extract, it is likely that the four-week period was insufficient. This was also confirmed by the study result that the fatigue recovery effect was higher on the fourth week than on the second week. 

The human placental extract is preferred, as it is known to be free from adverse events. As shown in this study, no serious adverse event was found in the human placental extract, and the frequency of its adverse events was also low. Thorough management and regulation of raw material collection and manufacturing are required, however, as the human placental extract is extracted from body tissues. 

Previous clinical studies on the human placental extract were mainly conducted to investigate hepatic dysfunction [10], menopausal disorder [11], and skin whitening [5, 6]. This study is valuable as a multicenter, double-blind study that first assessed the efficacy and safety of the oral human placental extract on fatigue. Furthermore, the results of this study are valuable because they can provide more treatment options to fatigue patients in the current situation wherein only a few effective treatments for fatigue are available. 

## 5. Conclusions

The oral human placental extract was effective in the improvement of fatigue. The adverse event frequency in the experimental groups was similar to that in the placebo group.

It is likely that more comprehensive results that can address the limitations of this study can be obtained if a study that has a sufficient period of study process and precisely classifies the subjects' ages and degrees of fatigue is conducted.

## Figures and Tables

**Table 1:** Schedule of the clinical trials.

	Screening	Treatment			Follow-up^3^
	Visit 1	Visit 2	Visit 3	Visit 4	Visit 5
	−2 weeks	1 day	2 weeks	4 weeks	6 weeks

Laboratory test^1^	○			○	○
Electrocardiogram	○				○
Pregnancy test^2^	○			○	
Physical examination & Vital signs	○	○	○	○	○
HADS	○				
CIS	○	○	○	○	○
Assessment of serious life events	○	○	○	○	○
Random assignment		○			
Adverse event			○	○	○

^1^Items of the laboratory test

(i) Blood test: CBC, ESR.

(ii) Serum biochemical assay: Glucose, BUN, Creatinine, Total protein, Albumin, Total bilirubin, AST, ALT, ALP, Total cholesterol, triglyceride, LDL-C, HDL-C.

(iii) Urine test: Protein, Glucose, Blood, WBC, pH.

^2^Conducted only on fertile women.

^3^Conducted only if adverse events were identified.

**Table 2:** Demographic information and characteristics of the subjects.

	Demographic information		Group 1	Group 2	Placebo group	*P *value
			*N* = 101	*N* = 102	*N* = 96	
Sex	Male	*n* (%)	13 (12.87)	22 (21.57)	13 (13.54)	0.1729^†^
	Female	*n* (%)	88 (87.13)	80 (78.43)	83 (86.46)	
Fertility	No	*n* (%)	24 (27.27)	16 (20.00)	16 (19.28)	0.3796^†^
	Yes	*n* (%)	64 (72.73)	64 (80.00)	67 (80.72)	

Age	Age (years)	Mean ± SD	41.20 ± 10.89	40.53 ± 11.23	39.97 ± 11.94	0.7486^$^
	18–29 years	*n* (%)	14 (13.86)	15 (14.71)	21 (21.88)	0.5497^†^
	30–39 years	*n* (%)	30 (29.70)	41 (40.20)	30 (31.25)	
	40–49 years	*n* (%)	33 (32.67)	25 (24.51)	24 (25.00)	
	50–59 years	*n* (%)	19 (18.81)	14 (13.73)	15 (15.63)	
	60–75 years	*n* (%)	5 (4.95)	7 (6.86)	6 (6.25)	

	Anxiety (HADS)	Mean ± SD	6.75 ± 2.59	5.81 ± 2.36	5.97 ± 2.55	0.0178^$^
	Depression (HADS)	Mean ± SD	6.74 ± 2.43	6.60 ± 2.41	6.69 ± 2.40	0.9114^$^
	Height (cm)	Mean ± SD	159.70 ± 6.50	161.20 ± 6.79	161.30 ± 7.10	0.1759^$^
	Weight (kg)	Mean ± SD	57.09 ± 9.19	57.73 ± 9.02	58.55 ± 9.54	0.5410^$^
	Heart rate (beat/minute)	Mean ± SD	73.94 ± 8.39	74.67 ± 8.76	74.69 ± 8.18	0.7759^$^
	Systolic BP (mmHg)	Mean ± SD	117.64 ± 13.64	115.44 ± 12.46	119.10 ± 13.26	0.1424^$^
	Diastolic BP (mmHg)	Mean ± SD	74.33 ± 9.81	72.78 ± 8.78	75.07 ± 9.46	0.2135^$^

Alcohol	No	*n* (%)	81 (80.20)	83 (81.37)	77 (80.21)	0.9710^†^
consumption	Yes	*n* (%)	20 (19.80)	19 (18.63)	19 (19.79)	
Serious life	No	*n* (%)	98 (97.03)	95 (93.14)	94 (97.92)	0.2544^‡^
events	Yes	*n* (%)	3 (2.97)	7 (6.86)	2 (2.08)	

^$^ANOVA, ^†^Chi-square test, ^‡^Fisher's exact test.

**Table 3:** Fatigue recovery rate of the CIS score.

Fatigue recovery rate		Group 1	Group 2	Placebo	*P *value^1^	*P *value^2^
		(*N* = 100)	(*N* = 99)	(*N* = 95)		
Visit 3	*n* (%)	51 (51.00)	58 (58.59)	44 (46.32)	0.5130^†^	0.0871^†^
Visit 4	*n* (%)	71 (71.00)	71 (71.72)	42 (44.21)	0.0002^†^	0.0001^†^

^†^Chi-square test, Cutoff score for fatigue recovery: CIS score of less than 76 points, ^1^Comparison of group 1 with the placebo group, ^2^Comparison of group 2 with the placebo group.

**Table 4:** CIS score change.

	Group 1 (*N* = 100)	Group 2 (*N* = 99)	Placebo group (*N* = 95)	*P *value^1^	*P *value^2^
	Mean ± SD	Mean ± SD	Mean ± SD		
Baseline (Visit 1)	92.54 ± 11.55	92.43 ± 10.85	91.84 ± 9.73	0.6495*	0.6899*
Visit 3	76.19 ± 12.96	71.49 ± 15.06	76.75 ± 14.60		
Visit 4	67.75 ± 13.49	68.02 ± 15.16	74.73 ± 16.45		
Baseline-Visit 3	−16.35 ± 14.38	−20.94 ± 16.09	−15.09 ± 15.27	0.5551*	0.0102*
*P *value	<0.0001^∫ ^	<0.0001^∫ ^	<0.0001^∫ ^		
Baseline-Visit 4	−24.79 ± 15.27	−24.41 ± 17.57	−17.12 ± 17.23	0.0012*	0.0039*
*P *value	<0.0001^∫ ^	<0.0001^∫ ^	<0.0001^∫ ^		

*Unpaired *t*-test, ^∫ ^Paired *t*-test, Visit 3 over the baseline = Visit 3, Baseline, Visit 4 over the baseline = Visit 4, Baseline, ^1^Comparison of group 1 with the placebo group, ^2^Comparison of group 2 with the placebo group.

**Table 5:** Rate of the CIS score reduction.

CIS score Reduction		Group 1 (*N* = 100)	Group 2 (*N* = 99)	Placebo (*N* = 95)	*P *value^1^	*P *value^2^
Yes	*n* (%)	80 (80.00)	76 (76.77)	59 (62.11)	0.0058^†^	0.0265^†^
No	*n* (%)	20 (20.00)	23 (23.23)	36 (37.89)		

^†^Chi-square test, Cutoff score for the reduction: CIS score (Baseline-Visit 4) >12 points, ^1^Comparison of group 1 with the placebo group, ^2^Comparison of group 2 with the placebo group.

**Table 6:** Summary of adverse events.

Adverse Event		Group 1 *N* = 101	Group 2 *N* = 102	Placebo group (*N* = 96)	*P *value
Adverse event (AE)	*n* (%)	19 (17.92)	16 (15.69)	16 (16.67)	0.9201^†^
Serious adverse event (SAE)	*n* (%)	1 (0.99)	0 (0.00)	0 (0.00)	0.6599^‡^
Adverse drug reaction (ADR)	*n* (%)	9 (8.91)	9 (8.82)	4 (4.17)	0.3477^†^
Withdrawal due to adverse events	*n* (%)	1 (0.99)	2 (1.96)	0 (0.00)	0.7758^‡^

^†^Chi-square test, ^‡^Fisher's exact test.
